# Endocrine manifestations and management of Prader-Willi syndrome

**DOI:** 10.1186/1687-9856-2013-14

**Published:** 2013-08-21

**Authors:** Jill E Emerick, Karen S Vogt

**Affiliations:** 1 Department of Pediatrics, Walter Reed National Military Medical Center Bethesda, 8901 Wisconsin Ave, Bethesda, MD 20889, USA

**Keywords:** Prader-Willi syndrome, Nutrition, Growth Hormone, Hypogonadism, Adrenal Insufficiency, Hypothyroidism, Glucose Abnormalities

## Abstract

Prader-Willi syndrome (PWS) is a complex genetic disorder, caused by lack of expression of genes on the paternally inherited chromosome 15q11.2-q13. In infancy it is characterized by hypotonia with poor suck resulting in failure to thrive. As the child ages, other manifestations such as developmental delay, cognitive disability, and behavior problems become evident. Hypothalamic dysfunction has been implicated in many manifestations of this syndrome including hyperphagia, temperature instability, high pain threshold, sleep disordered breathing, and multiple endocrine abnormalities. These include growth hormone deficiency, central adrenal insufficiency, hypogonadism, hypothyroidism, and complications of obesity such as type 2 diabetes mellitus. This review summarizes the recent literature investigating optimal screening and treatment of endocrine abnormalities associated with PWS, and provides an update on nutrition and food-related behavioral intervention. The standard of care regarding growth hormone therapy and surveillance for potential side effects, the potential for central adrenal insufficiency, evaluation for and treatment of hypogonadism in males and females, and the prevalence and screening recommendations for hypothyroidism and diabetes are covered in detail. PWS is a genetic syndrome in which early diagnosis and careful attention to detail regarding all the potential endocrine and behavioral manifestations can lead to a significant improvement in health and developmental outcomes. Thus, the important role of the provider caring for the child with PWS cannot be overstated.

## Introduction

Prader-Willi syndrome (PWS) is a genetic disorder, with a prevalence of 1/10,000-1/30,000, resulting from lack of expression of genes on the paternally inherited chromosome 15q11.2-q13. DNA methylation analysis within the Prader-Willi critical region detects the condition >99% of the time and is the first-line genetic test [1]. Further testing is required to determine the genetic subtype. Deletion of the paternally inherited 15q11.2-q13 region accounts for 70% of cases and can be determined by fluorescent in situ hybridization (FISH) or microarray. Maternal uniparental disomy (UPD) of chromosome 15 accounts for 25% of cases and can be determined by DNA polymorphism analysis in the affected individual and both parents. Imprinting defects account for most of the remaining 5% [2].

Major clinical manifestations of PWS include hypotonia with weak suck and poor feeding in infancy leading to failure-to-thrive, with later development of hyperphagia. Other clinical features include developmental delay, cognitive disability, and behavior problems, specifically stubbornness, obsessive-compulsive behaviors, and skin picking. Many of the clinical manifestations can be explained by hypothalamic dysfunction, including hyperphagia, temperature instability, high pain threshold, and sleep disordered breathing. The hypothalamic dysfunction also gives rise to variable pituitary hormone insufficiencies.

With improved recognition and availability of testing methodologies, PWS is being diagnosed earlier, often in the first few months of life. Earlier diagnosis, allowing for earlier access to developmental resources, recombinant human growth hormone (hGH) therapy, and anticipatory guidance, has significantly improved the long-term health and developmental outcomes of children with PWS [3]. Several excellent clinical guidelines have been published on the comprehensive management of PWS [3-5]. In this review, we focus on current evidence regarding the endocrine manifestations and their management with the goal of optimizing long-term outcomes for children and adolescents with PWS.

## Review

### Appetite and nutritional management

The transition from poor feeding and failure-to-thrive to hyperphagia is complex with 7 nutritional phases characterized by Miller et al. [6] (Table 1). Eating behavior and functional MRI studies have identified decreased satiety and increased reward response to food, in contrast to increased hunger, as major factors contributing to the hyperphagia [7-11]. The orexogenic hormone ghrelin is very high in individuals with PWS, even before the hyperphagia occurs [12,13]. However, decreasing ghrelin levels in these subjects with somatostatin or long-acting octreotide does not have an effect on appetite [14,15]. Lean body mass (LBM) is also decreased, resulting in decreased total and resting energy expenditure (REE), further promoting weight gain. [16].

**Table 1:** **Nutritional phases in Prader-Willi Syndrome**[6]

**Phase**	**Description**	**Median Age of Onset and Completion**
0	Decreased fetal movement and lower birth weight	In utero
1a	Hypotonia with difficulty feeding	0-9 months
1b	No difficulty feeding and growing appropriately	9-25 months
2a	Weight increasing without increase in appetite or excessive calories	2.1-4.5 years
2b	Weight increasing with an increase in appetite	4.5-8 years
3	Hyperphagic, rarely feels full	8 years - adulthood
4	Appetite no longer insatiable	adulthood

A strictly controlled diet and food security, both physical and psychological, are critical in the management of PWS. When children with PWS feel secure around food, their overall stress and anxiety are reduced and behavior is improved. Physical food security consists of locking up food and other physical barriers to food access. Principles of psychological food security include “No Doubt, No Hope (No Chance), and No Disappointment”. There is “no doubt” that the next meal is coming, nor how much and what type of food will be provided. The meal plan is known ahead of time and a structured routine is followed that focuses on the flow of activities rather than specific times. There is “no hope (no chance)” of food acquisition outside of what is provided, and there is “no disappointment” as what is promised is followed through and there are no false expectations [17].

Early dietary intervention and long-term nutrition monitoring lead to better outcomes. In one study, use of a controlled prescribed diet, starting at a median age of 14 months, resulted in normal body mass index (BMI) at age 10 in all subjects. The diet consisted of 10 kcal/cm length per day with a healthy balance of macronutrients. Interestingly, parents rarely reported that their children experienced hyperphagia or food cravings, suggesting a learned behavioral component of the hyperphagia [18]. A diet with carbohydrate content as low as 45% of total calories may have more favorable effects on body composition and fat utilization [19]. Prescribed daily physical activity is also important and contributes to improved body composition and REE [20].

### Growth hormone

The reported prevalence of growth hormone (GH) deficiency in PWS ranges from 40-100% depending on the diagnostic criteria used, with most studies reporting a prevalence in the higher end of this range [21,22]. Clinical manifestations consistent with GH insufficiency include short stature despite obesity, abnormal body composition, low insulin-like growth factor 1 (IGF-1) levels, and decreased GH secretion on provocative testing [23]. Even infants and toddlers, despite an often normal BMI, have increased fat mass and decreased LBM compared to normal children of the same age [24,25]. Recombinant human growth hormone (hGH) was FDA-approved in the United States in 2000 for the indication of “growth failure due to Prader-Willi syndrome.” In Europe, “improvement of body composition” is included in the approved indication of hGH therapy in PWS. In practice, hGH is primarily used for benefits other than increased height, including improved body composition and motor function. A consensus guideline on hGH therapy in PWS was recently published [23].

The longest term controlled study of hGH in PWS compared a cohort of children aged 6–9 years who had been treated with hGH for 6 years, starting at age 4–20 months, to a cohort of age and gender-matched children who had not been treated [26]. The treated group had significantly lower body fat percentage (36% versus 45%), greater muscle mass, more favorable lipid profiles, and better motor strength and function. Randomized controlled trials of hGH therapy from 12 to 30 months duration in infants as young as 4 months, toddlers, and older children have also shown significant improvements in body composition and height, with the greatest magnitude of change seen in the first year of therapy [27-33]. Improvements have also been reported in fat utilization, inspiratory muscle forces, motor function, hand and foot size, and lipid profiles in studies up to 4 years duration [27,34,35]. Cumulative hGH dosage over 10 years has correlated inversely with total body fat percentage [36].

Randomized controlled trials of hGH therapy for 1 and 2 years duration have also shown benefits on development and cognition. In one study, patients started on hGH prior to age 18 months had a significant increase in mobility scores on developmental testing [30]. Significant improvement in cognitive and motor development on the Bayley Scales of Infant Development II has been reported with hGH therapy, with those infants having the lower baseline motor skills showing the greatest improvement [31]. In another study, infants and toddlers progressed significantly more in language and cognitive development as assessed by the Capute scales [32]. In a 2 year randomized controlled trial of hGH in prepubertal children, intelligent quotient (IQ) standard deviation score (SDS) decreased in the non-treated group and remained stable in the treated group. IQ SDS then increased over 4 years when all individuals in the study were treated [37]. Proposed mechanisms for these observed improvements in cognition include positive effects of hGH on brain development, suggested by an association with head circumference growth, and an increased ability to interact with the learning environment due to improved motor function [31,32,37]. The effect size of these reported improvements however are relatively small so larger long-term studies are needed to further investigate this area.

One study investigating the effect of hGH on behavior in PWS showed improvement in depressive symptoms with the greatest improvement in those over 11 years old [38]. No deterioration in other aspects of behavior was found with hGH in this study, consistent with findings of other studies [39]. Furthermore, hGH had no effect on bone density after 2 years of treatment in prepubertal children and in adults with PWS [40,41].

The optimal age to start hGH is not known, but expert consensus is to start prior to the onset of obesity, which often occurs by age 2 [23]. Some experts recommend treating as early as 3 months of age[23]. Clinical guidelines recommend a starting dose of 0.5 mg/m^2^/day with progressive increase to 1 mg/m^2^/day [4,5,23]. A randomized controlled trial of hGH dosing showed that a dose of at least 1 mg/m^2^/day is required for positive effects on body composition [42]. Furthermore, most of the studies detailed above showing benefits of hGH used a dosage of 1 mg/m^2^/day.

The benefits of hGH therapy in childhood may persist into adulthood even after the hGH is discontinued. In one study, adults (mean age 25.4 years) who were treated with hGH during childhood had improved body composition and metabolic status compared to those who were not treated. The treated group had a lower mean BMI (32.4 versus 41.2), a greater percentage with BMI <30 (45% versus 18.2%), lower mean hemoglobin A1c, lower mean insulin resistance index, and less hypertension. The treated group had been diagnosed at a younger age (4.8 versus 10.1 years) so other aspects of the earlier diagnosis may have contributed to the improved outcomes [43].

The role of hGH therapy in adults with PWS is less clearly defined. Recent studies have begun to illuminate the risk benefit ratio of hGH treatment in this patient population. The prevalence of GH deficiency in adults with PWS ranges from 15% to 95%, depending on the agents used for stimulation testing and the threshold GH level used to define deficiency [44,45]. The average reported prevalence of severe GH deficiency is 40-50% [46]. Those with the deletion subtype have higher stimulated GH responses than those with the UPD subtype [47]. Beneficial effects of hGH therapy in adults with PWS when administered for 6 months to several years include decreased fat mass, increase in LBM, and improved respiratory muscle function [44-46]. In several studies, edema was reported after initiation of hGH, but not to a degree that led to cessation of therapy [46]. Evidence is conflicting on the degree that hGH therapy affects fasting glucose, fasting insulin and homeostatic model assessment (HOMA) index but close monitoring of glucose homeostasis in treated patients is warranted [44-46]. Many regulatory agencies require dynamic testing to diagnose GH deficiency prior to treating adults with PWS [23]. However, there is currently no consensus on the need for such testing after the attainment of adult height as those who do not test GH deficient may also benefit from hGH therapy. Further investigation is needed in this area. Recent expert consensus recommends a starting dose of 0.1-0.2 mg/day in adults with maintenance of IGF-1 levels between 0 and + 2 SDS to achieve beneficial effects of hGH with the lowest possible risk for adverse events [23].

HGH therapy in PWS is not without risk and needs to be undertaken thoughtfully. Contraindications according to the pharmaceutical companies and expert consensus include severe obesity, untreated severe obstructive sleep apnea (OSA), uncontrolled diabetes, active malignancy, and active psychosis [23]. Concerns have been raised regarding the association of hGH with excessive elevations in IGF-1, sleep disordered breathing, scoliosis, alterations in glucose metabolism, and sudden death. There is an increasingly recognized phenomenon of high IGF-1 levels despite relatively low hGH doses that seems to be unique to PWS. Potential concerns related to excessively high IGF-1 levels include lymphoid hyperplasia leading to OSA and a theoretical increase in malignancy risk. In a study of 55 children with PWS treated with GH for 4 years, IGF-1 levels increased significantly in the first year of therapy and decreased slightly at year 4 (mean SDS +2.1). Three subjects had IGF-1 levels >3.5 SDS that declined to 2–3 SDS after GH dose reduction [35]. In one study, IGF-1 and IGF binding protein 3 (IGFBP3) levels were evaluated over a 2 year period in a group of 33 children with PWS treated with hGH. These subjects were compared to 591 subjects treated for GH deficiency. The PWS group had significantly higher IGF-1 levels despite lower doses of hGH. However, there was no significant difference in IGF-1 to IGFBP3 molar ratios between groups, suggesting that bioavailable IGF-1, and therefore risk for adverse effects, may be similar in both groups [48]. The optimal management of these high IGF-1 levels is currently unclear. The potential risk needs to be balanced against the evidence that at least 1 mg/m^2^/day is required for favorable effects on body composition. Current recommendations are to monitor IGF-1 levels at least every 6 to 12 months and attempt titrate the hGH dosage to maintain levels between +1 and +2 SDS [23].

Patients with PWS have a high incidence of both central and obstructive sleep apnea [49-51]. Factors contributing to the sleep disordered breathing include obesity, restrictive lung disease due to muscle weakness or scoliosis, reduced ventilatory response to hypercapnia, and hypoxia during sleep and wakefulness [52]. HGH therapy potentially worsens sleep disordered breathing because increased IGF-1 levels lead to lymphoid hyperplasia [53,54]. One study showed decreased central sleep apnea in patients treated with hGH, but worsening of OSA that correlated with elevated IGF-1 levels [53]. In other studies, hGH therapy was also associated with beneficial effects on central aspects of sleep disordered breathing, as well as inspiratory and expiratory muscular strength [34,39].

Current guidelines recommend the following evaluation of sleep disordered breathing prior to starting hGH: 1). Otolaryngology (ENT) referral if there is a history of sleep disordered breathing, snoring, or if enlarged tonsils and adenoids are present, with consideration of tonsillectomy and adenoidectomy. 2). Referral to a pulmonologist or sleep clinic. 3). Sleep oximetry in all patients, preferably by polysomnographic evaluation. Significant OSA should be treated prior to starting hGH. Repeat polysomnography is recommended within the first 3–6 months of starting hGH [23].

Due in part to the underlying hypotonia, scoliosis affects 30-80% of patients with PWS [55]. Multiple studies, including randomized controlled trials, have shown no effect of hGH therapy on scoliosis, even in patients started on hGH at younger ages [26,27,56-58]. Scoliosis is not considered a contraindication for initiating or continuing hGH therapy in patients with PWS. However, prior to initiating therapy, a spine film with orthopedic referral if necessary is recommended. Once hGH therapy is initiated, a spine film and/or orthopedic assessment should be considered if there is concern about scoliosis progression [23].

Alteration in glucose metabolism is another side effect to consider in patients with PWS receiving hGH. HGH can lead to increased insulin resistance due to its counter-regulatory effects on insulin action. Pediatric studies show no significant alterations in glucose homeostasis with hGH therapy for up to 4 years [26,27,35]. Adult studies show a minor increase in fasting glucose and a trend toward increased fasting insulin and HOMA, but no change in hemoglobin A1c [46]. Recent expert consensus recommends surveillance of hemoglobin A1c, fasting glucose, and fasting insulin for those receiving hGH therapy and consideration of an oral glucose tolerance test (OGTT) for those who are obese, and/or >12 years old, and/or have a family history of diabetes [23].

The association of hGH therapy with sudden death in PWS has received significant attention. Between 2002 and 2006, 20 deaths were reported in children with PWS treated with hGH, but evidence has not been convincing that there is a causative relationship between sudden death and hGH therapy [59]. In a review of 64 cases of death in children with PWS ranging in age from a few days of life to 19 years, 28 subjects (44%) were receiving hGH therapy at the time of death. Respiratory disorders were the most common cause of death, and there were no differences in cause of death between the hGH treated and untreated group. However, 75% of the deaths in the hGH treated group occurred within 9 months of hGH therapy initiation, a finding that suggests a need for close surveillance for any exacerbation of sleep related breathing disorders during the first year of hGH therapy [60]. Sudden death and the possible association with hGH therapy may be related to the increased risk for central adrenal insufficiency (see below), especially in the setting of an acute respiratory illness [60].

### Adrenal insufficiency

Central adrenal insufficiency occurs in PWS but the frequency is unclear. Children and adults with PWS are at risk for adrenal insufficiency due to the generalized hypothalamic dysfunction. In a case series of unexpected death in PWS, autopsies performed in 3 out of 4 young children who presented with febrile or other acute illnesses revealed small adrenal glands by weight criteria. The 4th child’s adrenal weight was below average [61]. Also reported is an adolescent male with PWS who developed symptomatic adrenal insufficiency during spine surgery that resolved promptly after administration of glucocorticoid [62]. None of these patients were reported to have been on hGH. GH inhibits 11β-hydroxysteroid dehydrogenase type 1 (11βHSD-1), resulting in less cortisone conversion to active cortisol. Thus hGH may further blunt the stress response to an acute illness when the hypothalamic-pituitary-adrenal axis is already not functioning optimally. The first published cross-sectional analysis (n = 25) of adrenal insufficiency in children with PWS found that 60% tested insufficient by overnight single-dose metyrapone testing. Baseline hormone levels were not different in those who tested sufficient versus insufficient suggesting that there is a deficit in the reserve necessary for the stress response [63]. Subsequent studies using different testing methodologies, including low-dose and high-dose Synacthen and the insulin tolerance test, did not show the same frequency of adrenal insufficiency with the highest percentages at 14-15% [64-68]. These studies are summarized in Table 2. The true prevalence of central adrenal insufficiency in PWS remains unclear and is an area in need of further investigation.

**Table 2:** Studies on Adrenal function in Prader-Willi syndrome

**Study**	**N**	**Mean age, range (years)**	**Test**	**% Insufficient**	**Comment**
De Lind van Wijingaarden 2008 [63]	25	9.7 (3.7-18.6), median	Overnight Single-dose Metyrapone	60	Baseline salivary cortisol levels not significantly different in sufficient versus insufficient
Grugni 2013 [64]	53	27.9 (18–45.2)	LDSST	15.1	
Corrias 2012 [65]	84	7.7 (0.8-17.9)	LDTST	14.2	Lower cortisol peak with deletion subtype versus UPD
Connell 2010 [66]	25	7.2 (0.43-16.3)	ITT (15), HDSST (10), LDSST (4)	4	1 subject tested insufficiency by ITT
Nyunt 2010 [67]	41	7.7	LDSST	0	
Farholt 2011 [68]	57	22 (0.58-48)	HDST	0	
Farholt 2011 [68]	8	25 (16–33)	ITT	0	

LDSST = low-dose short Synacthen test (1 μg).

LDTST = low-dose tetracosactrin stimulation test (1 μg).

HDSST = high-dose short Synacthen test (250 μg).

UPD = uniparental disomy.

HDST = high-dose Synacthen test (250 μg).

ITT – insulin tolerance test (0.15 units/kg).

As a result, no consensus exists on the appropriate evaluation and management of central adrenal insufficiency in PWS. Obtaining cortisol and ACTH levels during an acute illness or other stressful situation may provide useful diagnostic information. One group recommends considering stress-dose steroids for all patients with PWS during stress, to include mild upper respiratory infections, since patients with PWS often do not show significant signs of illness such as fever or vomiting [63]. Another group recommends considering prophylactic stress dose steroids for major surgery or at least having them readily available to administer for symptoms of adrenal insufficiency [62]. In our practice, we discuss with all families the possibility of adrenal insufficiency during stress and provide our patients with stress doses of hydrocortisone to store at home for administration during significant illness. We also recommend peri-operative stress dose steroids.

### Hypogonadism

Hypogonadism is a consistent feature of both males and females with PWS. Clinical presentation includes genital hypoplasia, delayed or incomplete puberty, and infertility in the vast majority. Genital hypoplasia is evident at birth. In females it manifests as clitoral and labia minora hypoplasia and can easily be overlooked on physical examination. Males commonly have cryptorchidism, a poorly rugated, under pigmented, hypo plastic scrotum, and may have a small penis. Unilateral or bilateral cryptorchidism is present in 80-90% of males [2]. One author recommends considering a trial of human chorionic gonadotropin (hCG) to promote testicular descent for potential avoidance of surgical correction and general anesthesia given the risk for respiratory complications. HCG may also increase scrotal size and penile length, which can improve orchidopexy outcomes and facilitate later standing micturition [3]. However, there are no published data regarding the efficacy of this practice in patients with PWS. Surgical correction of cryptorchidism should be completed in the first or second year of life [3,4].

Hypogonadism was classically thought to be hypothalamic in etiology, similar to many other manifestations of PWS. However, recent evidence has emerged supporting primary gonadal failure as a significant contributor to male hypogonadism [69-71]. A recent longitudinal study of gonadal function in 68 males with PWS ages 6 months to 16 years showed that inhibin B levels were normal in the prepubertal period, but decreased significantly with a concordant rise in follicle-stimulating hormone (FSH) after puberty onset. Testosterone levels increased during puberty but remained below the 5th percentile, while luteinizing hormone (LH) levels increased but not above the 95% [70]. Other studies have also shown a combined picture of hypogonadotropic hypogonadism with relatively low LH levels, and primary hypogonadism with low inhibin B and relatively high FSH levels [71,72]. Gonadal function has also been evaluated longitudinally in 61 girls with PWS. The primordial follicle pool and the number of small antral follicles were conserved. However, maturation of follicles and progression of pubertal development were impaired. LH levels were relatively low for the low estradiol levels observed, and FSH levels were normal. Pubertal onset was similar in timing to the normal population, but progression was delayed [73].

Although most patients with PWS present with delayed and/or incomplete puberty, other pubertal variations have been reported. Precocious adrenarche occurs in 15-30% of patients and is felt to be secondary to obesity or possibly increased adrenal exposure to insulin or IGF-1 [74]. Precocious puberty has been reported in 4% of both boys and girls [74-76]. Treatment of precocious puberty with gonadotropin releasing hormone (GnRH) analogs is not indicated as pubertal advancement is not sustained [4].

Many patients with PWS require hormonal treatment for induction, promotion or maintenance of puberty. Benefits of sex steroid replacement include positive effects on bone health, muscle mass, and possibly general well-being. No consensus exists as to the most appropriate regimen for pubertal induction or promotion but experts agree that the dosing and timing should reflect as closely as possible the process of normal puberty [4]. Available data suggest that sex steroid deficiency contributes to low bone density in adults with PWS [77,78]. Therefore, in females, sex hormone replacement should be considered if there is amenorrhea/oligomenorrhea or low bone mineral density (BMD) in the presence of reduced estradiol levels [77]. Testosterone administration should be considered in males with PWS as for any other hypogonadal patient. Androgen therapy can be more physiologically administered using testosterone patches and gel preparations. These delivery systems avoid the peaks and troughs of injections, which may be of particular importance in PWS because of historical concerns about aggressive behaviors with testosterone treatment [4]. However, patients may have difficulty with topical treatment due to skin irritation and skin picking behaviors.

No cases of paternity have been reported in PWS, but 4 pregnancies have been documented in females with PWS. These 4 reported pregnancies resulted in 2 normal offspring and 2 offspring with Angelman Syndrome [79,80] (unpublished abstract, Cassidy SB and Vats D, 25th annual Prader-Willi Syndrome Association scientific meeting in Orlando, FL Nov 2011). The potential for fertility in females with PWS necessitates discussion of sexuality and birth control at an appropriate age.

### Hypothyroidism

Hypothyroidism has been reported in approximately 20-30% of children with PWS [22,81]. Similar to other endocrinopathies in PWS, the etiology is thought to be central in origin. A recent study of children with PWS under 2 years of age revealed that 72.2% had abnormalities in the hypothalamic-pituitary-thyroid axis evidenced by low total or free thyroxine (FT4 ) in the presence of normal thyroid stimulating hormone (TSH) [82]. Studies of adult patients with PWS show that the frequency of thyroid disease is 2%, which is similar to that of the general population [83]. In a study of thyroid function in 75 children with PWS treated with hGH therapy at 1 mg/m^2^/day for 1 year, FT4 levels dropped significantly, while triiodothyronine (T3) levels did not change, consistent with increased GH-promoted peripheral conversion of T4 to T3 [84]. Studies investigating the natural history of hypothyroidism in patients with PWS, as well as the effects of thyroid hormone treatment, are needed. We recommend that FT4 and TSH be screened in the first 3 months of life and annually thereafter, especially if the patient is receiving hGH therapy, with initiation of levothyroxine if hypothyroxinemia is discovered.

### Glucose metabolism and diabetes

Type 2 diabetes mellitus has been reported in 25% of adults with PWS with onset at a mean age of 20 years. The mean BMI of those who developed type 2 diabetes in this cohort was 37 kg/m^2^[85]. Diabetes and impaired glucose tolerance are much less frequent in children with PWS. A study of 74 children with PWS at a median age of 10.2 years showed that none had type 2 diabetes and only 4% had impaired glucose tolerance by OGTT [22]. Several studies have demonstrated that subjects with PWS, not receiving hGH therapy, had lower insulin levels and greater insulin sensitivity compared to controls matched for degree of obesity [86,87]. Proposed reasons for the increased insulin sensitivity in PWS include relative diffuse as opposed to visceral obesity, lower GH levels, and higher ghrelin levels for the degree of obesity [88,89]. Periodic surveillance for diabetes and features of the metabolic syndrome should be undertaken in obese individuals as is recommended for obesity in the general population. Similarly, evaluation of diabetes risk is recommended prior to initiation of hGH in obese patients over 12 years of age, with periodic surveillance for those on hGH treatment [23].

## Conclusions

Early diagnosis and comprehensive care of patients with PWS have improved outcomes. Table 3 summarizes the screening and management of the endocrine manifestations of PWS. Areas where further research is needed include the etiology and management of hyperphagia, risks and management of high IGF-1 levels associated with relatively low hGH doses, optimal surveillance of sleep disordered breathing, further elucidation of the effect of hGH on cognition, the impact of hGH therapy in adulthood, frequency and management of adrenal insufficiency, and the frequency and natural history of hypothyroidism.

**Table 3:** Endocrine Management of patients with Prader-Willi syndrome

**Age**	**Area to Address**	**Testing/Treatment**
Birth to 3 months or at diagnosis	Diagnosis	• DNA methylation analysis as initial test
		• Subsequent determination of genetic subtype
	Hypothyroidism	• TSH, FT4
		• Start treatment if hypothyroxinemic
	Growth Hormone	• Initiate discussion of hGH therapy
3 months through childhood	Hyperphagia	• Provide education on: nutritional phases, need for food security, strict dietary control and routine, regular physical
		• Nutrition referral
	Cryptorchidism	• Urology referral
		• Consider trial of hCG
	Hypothyroidism	• Annual TSH and FT4 starting at age 1
	Growth Hormone	• Consider starting therapy in the first few months of life, or prior to onset of obesity.
		• No pre-treatment testing required.
		• Starting dose: 0.5 mg/m2/day with progressive increase to 1 mg/m2/day.
		• Aim to keep IGF-1 levels between +1 and +2 SDS
	Growth Hormone Monitoring	Prior to starting therapy:
		1. Otolaryngology referral if there is a history of sleep disordered breathing, snoring, or enlarged tonsils or adenoids are present, with consideration of tonsillectomy and adenoidectomy
		2. Referral to a pulmonologist or sleep clinic
		3. Sleep oximetry in all patients, preferably polysomnographic evaluation
		4. Spine film with orthopedic referral if significant scoliosis present
		5. Bone age film if at appropriate chronologic age
		6. Consider body composition evaluation (e.g. DXA)
		Contraindications to therapy:
		1.Untreated severe OSA
		2.Uncontrolled diabetes
		3.Severe obesity
		4.Active malignancy
		5.Active psychosis
		While on therapy:
		1. IGF-1 every 6–12 months
		2. Repeat polysomnography within the first 3–6 months of initiating hGH therapy
		3. Spine film and/or orthopedic assessment if concerns for scoliosis progression
	Adrenal insufficiency	• Consider obtaining cortisol and ACTH levels during acute illness or other stressful situation to clarify diagnosis
		• Consider stress dose steroids for all patients with PWS during stress, to include mild upper respiratory infections and the perioperative period
Puberty through adulthood	Hypogonadism	• Sex steroid therapy as needed to promote normal timing and progression of puberty in males and females
		• Adult females: sex steroid replacement if oligo/amenorrhea or low BMD in the setting of a low estradiol level
		• Adult males: testosterone replacement as for hypogonadal males. May be behavioral benefits from topical androgen formulations
	Growth Hormone	• Adults: evaluate the GH/IGF-1 axis prior to initiating hGH
		• Adult staring dose: 0.1-0.2 mg/day
		• Aim to keep IGF-1 0 to +1 SDS
	Diabetes	• Screen prior to initiation of and annually during growth hormone therapy in patients ≥ 12 years of age
		• Screen in obese individuals as is recommended for the general population
	Obesity	Periodic monitoring of/for:
		1. Lipid profiles
		2. Hepatic steatosis

Resources for families and providers can be found through the Prader-Willi syndrome Association USA (http://www.pwsausa.org), the Foundation for Prader-Willi Research (http://fpwr.org), and the International Prader-Willi syndrome Organization (http://www.ipwso.org).

## Abbreviations

ACTH: Adrenocorticotropic hormone; 11βHSD-1: 11β-hydroxysteroid dehydrogenase type 1; BMD: Bone mineral density; BMI: Body mass index; DNA: Deoxyribonucleic acid; ENT: Ears, nose, and throat; FDA: Food and drug administration; FISH: Fluorescent in situ hybridization; FSH: Follicle-stimulating hormone; FT4: Free thyroxine; GH: Growth hormone; GnRH: Gonadotropin releasing hormone; hCG: Human chorionic gonadotropin; hGH: Human growth hormone; HOMA: Homeostatic model assessment; IGF-1: Insulin-like growth factor 1; IGFBP3: Insulin-like growth factor binding protein 3; IQ: Intelligence quotient; LBM: Lean body mass; LH: Luteinizing hormone; MRI: Magnetic resonance imaging; OGTT: Oral glucose tolerance test; OSA: Obstructive sleep apnea; PWS: PRADER-Willi syndrome; REE: Resting energy expenditure; SDS: Standard deviation score; T3: Triiodothyronine; TSH: Thyroid stimulating hormone; UPD: Uniparental disomy.

## Competing interests

The authors declare that they have no competing interests.

## Authors’ contributions

JE contributed substantially to the writing of this manuscript. KV drafted the outline of the manuscript and contributed to the writing. Both authors read and approved the final manuscript.

## Authors’ information

JE is faculty in the Division of Pediatric Endocrinology at the Walter Reed National Military Medical Center Bethesda and is a CDR in the US Navy. She has a special interest in childhood and adolescent obesity. KV is Chief of Pediatric Endocrinology at Walter Reed National Military Medical Center Bethesda and is a LTC in the US Army. She has a 3 year-old son with Prader-Willi syndrome and is active in the Prader-Willi Syndrome Association USA and the Foundation for Prader-Willi Research.
